# A clinical screening algorithm for primary hyperoxaluria type 1 in adults on dialysis

**DOI:** 10.1093/ndt/gfad184

**Published:** 2023-09-14

**Authors:** Pietro Manuel Ferraro, Viola D'Ambrosio, Giovanni Gambaro, Daniela Giachino, Jaap Groothoff, Giorgia Mandrile

**Affiliations:** Section of Nephrology, Università degli Studi di Verona, Italy; U.O.S. Terapia Conservativa della Malattia Renale Cronica, Fondazione Policlinico Universitario A. Gemelli IRCCS, Rome, Italy; Università Cattolica del Sacro Cuore, Rome, Italy; Università Cattolica del Sacro Cuore, Rome, Italy; Medical Genetic Unit, San Luigi Gonzaga University Hospital, Torino, Italy; Department of Pediatric Nephrology, Emma Children's Hospital, Amsterdam UMC, University of Amsterdam, Amsterdam, The Netherlands; Genetic Unit and Thalassemia Center, San Luigi Gonzaga University Hospital, Torino, Italy


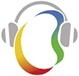
 Watch the video of this contribution at https://academic.oup.com/ndt/pages/author_videos

To the Editor,

Primary hyperoxaluria (PH) is a group of genetic disorders characterized by excessive production of oxalate by the liver [1]. Three forms are known, PH type 1 (PH1), the most common, due to mutations of the *AGXT* gene, type 2 (*GRHPR* gene) and type 3 (*HOGA* gene) [2]. In humans, oxalate is an end-product that is primarily excreted by the kidney through filtration and secretion [3]. The abnormal production of oxalate in patients with PH increases the urinary supersaturation for calcium oxalate, leading to formation of kidney stones and nephrocalcinosis, which in turn decrease plasma oxalate clearance [4]. PH leads in over 70% of cases to end-stage kidney disease (ESKD); with the progression of kidney damage, the concentration of oxalate in blood reaches the supersaturation point and calcium oxalate deposits in several organs; this picture is defined as ‘systemic oxalosis’ and carries significant mortality.

PH is generally considered an ultra-rare, but underdiagnosed PH condition, especially in the adult setting. A considerable number of PH patients are diagnosed after ESKD or kidney transplantation. The appearance of new RNAi-based oxalate-reducing therapies makes an early diagnosis even more relevant [5]. The aim of the work presented here is to develop and validate a prediction model for the diagnosis of PH1 in a population of adult patients undergoing haemodialysis (HD).

PH patients were derived from the database of genetically resolved Italian PH patients at the Genetics Unit of San Luigi Hospital (a national referral center for the genetic diagnosis of PH). For this study, only patients with an established genetic diagnosis of PH1 who underwent HD during adulthood were included. This database represents the Italian section of the OxalEurope Registry, and its characteristics have been previously described [6].

Non-PH patients were adult patients undergoing chronic HD at Fondazione Policlinico Universitario A. Gemelli IRCCS. Patients with an already established diagnosis of kidney disease were excluded. The assumption that no patient was affected by PH1 was tested by sequencing of the *AGXT* gene, through a buccal swab, in 45 patients.

For both groups, information on five pre-specified ‘red flags’ was systematically extracted from medical records: active nephrolithiasis, defined as recurrent and/or with early onset (before 25 years) and/or bilateral and/or with significant family history (defined as at least two subjects affected within the family); presence of nephrocalcinosis; previous graft loss; early initiation of HD (before 40 years of age); family history of chronic kidney disease (CKD).

Characteristics of patients included in the study were summarized by means and standard deviations or frequencies and percentages. Next, we divided our study population into a training sample (70% of the original sample) and a validation sample (30% of the original sample). In the training sample, a multivariable logistic regression model was applied to estimate the probability of PH1 as a function of the five ‘red flags’. A score (‘PH score’) was constructed based on the coefficients of the predictors included in the regression model and its performance in identifying PH1 was evaluated in terms of discrimination [area under the ROC curve (AUC) and 95% confidence intervals (CIs)] and calibration (predicted vs observed probability plots); the PH score was then applied to the validation sample and metrics of performance were calculated again. All analyses were performed with Stata 16.1 (StataCorp, College Station, TX, USA).

The final study population consisted of 107 patients, of whom 32 were PH1 and 75 non-PH patients (Table 1). The proportion of men was similar in the two groups, whereas PH1 patients initiated HD at a significantly younger age compared with non-PH patients (on average 38 vs 51 years). Overall, a history of active nephrolithiasis and early HD initiation were more common among PH1 patients, whereas a family history of CKD was not, as expected for a recessive disease. Since no patient in the non-PH group had nephrocalcinosis, this item was not considered further in modelling the probability of PH1 as it had to be dropped due to complete separation.

**Table 1:** Characteristics of the study population.

	PH1 group (*n* = 32)	Non-PH group (*n* = 75)
Age at HD initiation (years) (mean, SD)	38 (12)	51 (20)
Men (*n*, %)	21 (66)	45 (60)
Active nephrolithiasis (*n*, %)	26 (81)	10 (13)
Presence of nephrocalcinosis (*n*, %)	12 (38)	0
Previous graft loss (*n*, %)	7 (22)	9 (12)
Early initiation of HD (*n*, %)	20 (63)	15 (20)
Family history of CKD (*n*, %)	6 (19)	26 (35)

SD, standard deviation.

**Figure 1:**
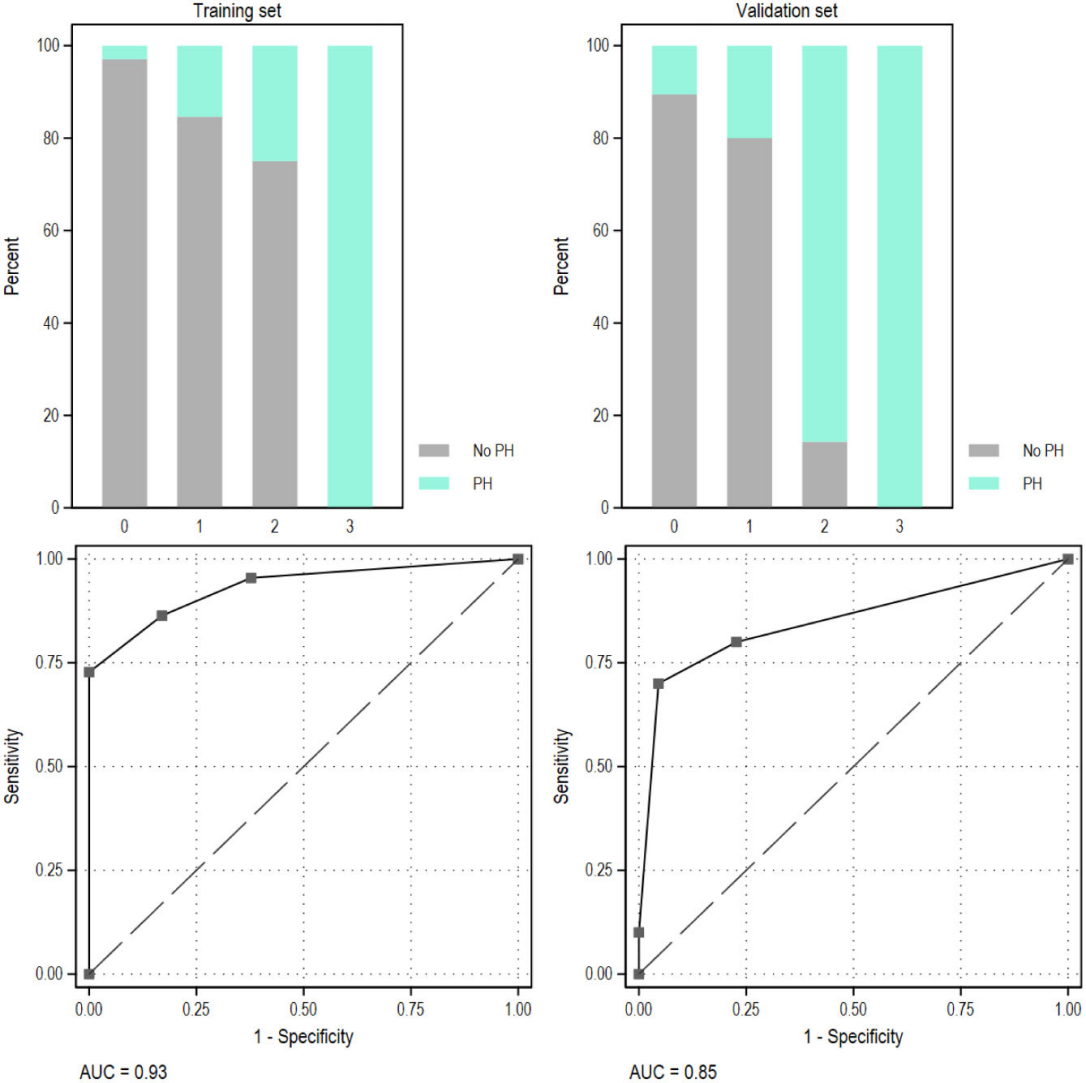
The dataset was split into a training (2/3) and a validation (1/3) set, distributions for each criterion were compared, and a logistic regression model was used to calculate a score reflecting the likelihood of PH1. Performance metrics including discrimination [area under the ROC curve (AUC)] and calibration (predicted vs observed plots) were computed on both sets.

The training set included 22 PH1 and 53 non-PH patients. The PH1 score based on the coefficients obtained from the logistic regression model had a range from 0 to 3. The discrimination of the model was high (AUC 0.93, 95% CI 0.86, 1.00), and the calibration was appropriate (Fig. 1, left).

The validation set included 10 PH1 and 22 non-PH patients. In those patients, the PH score based on the coefficients obtained from the training set confirmed its discrimination (AUC 0.85, 95% CI 0.69, 1.00) and calibration properties (Fig. 1, right).

We used five readily available clinical features, including active nephrolithiasis, presence of nephrocalcinosis, a previous graft loss, early initiation of HD and family history of CKD, to construct a prediction model whose output reflects the probability of PH1 in a setting of adult patients undergoing HD. Our prediction model showed good performance, with high discrimination and calibration even with external validation. Our model could be easily implemented in dialysis facilities to identify high-risk patients in whom a second-level investigation could be performed (such as obtaining further clinical information, plasma oxalate or sequencing of the genes involved in PH). Based on our results, different thresholds for action based on the PH score might be analysed to optimize sensitivity or specificity; for instance, in our dataset sensitivity and specificity were 91%/67%, 81%/87% and 53%/100% when moving the cutpoint from 1+ to 3. Since our model is intended as an inexpensive screening measure, we would recommend a low threshold for action (e.g. obtain further information in patients with a PH score of at least 1), especially for those patients being evaluated for a kidney transplant. An online PH score calculator is available at (https://qxmd.com/calculate/calculator_886).

PH is considered an ultra-rare disease, although recent estimates suggest a prevalence of about 1 in 60 000 individuals, and it is more frequent among individuals of European ancestry (1 in 40 000) [7]. About 80% of PH cases are represented by PH1, the most severe type, with a cumulative risk of developing ESKD at 30 years of 73% [8]. Even after initiation of HD, the prognosis of PH patients is abysmal due to the difficulty of eliminating excess oxalate production even with extensive dialysis schedules [9]. Although some mutations respond to administration of pyridoxine, only a minority of patients fully respond to this therapeutic approach [10]. Thus, until recently, a combined liver and kidney transplant was considered as the only curative strategy for patients affected by PH1 [11]. However, new pharmacological approaches are currently available or in development, which have proven effective in reducing urinary and blood oxalate levels in affected patients [12, 13]. In particular, real-world evidence is accumulating on the effectiveness and safety of lumasiran, the only drug currently approved for PH1, as a strategy to pursue isolated kidney transplant in dialysis patients [14]. This reinforces the need for a correct and timely diagnosis in affected patients, a target that might be achieved by our proposed tool. It should however be noted that other rare monogenic causes of kidney stones such as Dent's disease and cystinuria can lead to ESKD and should be considered in the differential diagnosis. Another advantage of our tool is the use of a small number of inexpensive and easy to obtain variables, which will allow the implementation of the algorithm at bedside and/or its automation using electronic health records. We are currently planning a prospective, multicentric study to estimate the diagnostic yield of our algorithm.

In conclusion, a simple algorithm implemented by easily accessible clinical parameters is a useful screening tool for the diagnosis of PH1 in adult patients undergoing dialysis.
